# Editor’s Book Table

**Published:** 1871-03

**Authors:** 


### Editor's Book Table.

[NOTE.—All works reviewed in the columns of the CHICAGO MEDICAL JOURNAL may be found in the extensive stock of W. B. KEEN & COOKE, whose catalogue of Medical Books will be sent to any address upon request.]

BOOKS RECEIVED.

*The Change of Life in Health and Disease:* A Practical Treatise on the Nervous and other Affections Incidental to Women at the Decline of Life. By Edward John Tilt, M.D., etc., etc. From the third London Edition. Philadelphia: Lindsay & Blakiston, 1871; pp. 292; $3.

*A Treatise on the Chronic Inflammation and Displacements of the Unimpregnated Uterus.* By William H. Byford, A.M., M.D., etc., etc. Second Edition, enlarged; with numerous Illustrations. Philadelphia: Lindsay & Blakiston, 1871; pp. 248; $3.

*First Medical Report of the Boston City Hospital.* Edited by J. Nelson Borland, Physician; David W. Cheever, Surgeon. Boston: Little, Brown & Company; 1870; pp. 688; with Illustrations.

### PAMPHLETS RECEIVED.

*American Association for the Cure of Inebriates.* Proceedings of the First Meeting, held in New York, November 29 and 30, 1870.

*New York State Inebriate Asylum, Binghampton, N. Y.* Annual Report of the Superintendent and Physician, for the year 1870.

*Braithwaite's Retrospect of Practical Medicine and Surgery:* Part LXII; January, 1871. Uniform American Edition. New York: W. A. Townshend & Adams, publishers; $2.50 per year; single No., $1.50.

*The Half-Yearly Abstract of the Medical Sciences.* Being a Digest of British and Continental Medicine, and of the Progress of Medicine and Surgery, and the Collateral Sciences. Edited by Wm. Domett Stone, M.D., F.R.C.S. (Exam.) Vol. LII, January, 1871. Philadelphia: Henry C. Lea, 1871; $2.50 per annum; single volumes, $1.50.

*Catarrhal and Croupous Inflammations of Mucous Membranes.* By Samuel G. Armor, M.D., Professor of Principles and Practice of Medicine, in Long Island College Hospital, Brooklyn, N. Y.

*On the Cellular Structure of the Red Blood Corpuscle.* By Joseph G. Richardson, M.D., Microscopist to the Pennsylvania Hospital, Philadelphia, Pa.

*Transactions of the American Ophthalmological Society.* 7th Annual Meeting. Newport: July, 1870; pp. 151.

No. 5. *University Series, Scientific Addresses.* By Professor John Tyndall, LL.D., F.R.S., Royal Institution: 1. On the Methods and Tendencies of Physical Investigation. 2. On Haze and Dust. 3. On the Scientific Use of the Imagination. New Haven, Conn.: Charles C. Chatfield & Co.; 1870; 12mo, 25 cts. series; nearly monthly. Five successive Nos., in advance, $1.10; ten successive Nos., in advance, $2.

